# Distinct chromophore–protein environments enable asymmetric activation of a bacteriophytochrome-activated diguanylate cyclase

**DOI:** 10.1074/jbc.RA119.011915

**Published:** 2019-12-04

**Authors:** David Buhrke, Geoffrey Gourinchas, Melanie Müller, Norbert Michael, Peter Hildebrandt, Andreas Winkler

**Affiliations:** ‡ Technische Universität Berlin, Institut für Chemie, Sekr. PC14, Straβe des 17. Juni 135, D-10623 Berlin, Germany; § Institute of Biochemistry, Graz University of Technology, Petersgasse 12/II, 8010 Graz, Austria; ¶ Max Planck Institute for Medical Research, Jahnstrasse 29, 69120 Heidelberg, Germany; ‖ BioTechMed-Graz, 8010 Graz, Austria

**Keywords:** photoreceptor, allosteric regulation, cyclic di-GMP (c-di-GMP), crystallography, infrared spectroscopy (IR spectroscopy), asymmetry, GGDEF, photoconversion, phytochrome, symmetry

## Abstract

Sensing of red and far-red light by bacteriophytochromes involves intricate interactions between their bilin chromophore and the protein environment. The light-triggered rearrangements of the cofactor configuration and eventually the protein conformation enable bacteriophytochromes to interact with various protein effector domains for biological modulation of diverse physiological functions. Excitation of the holoproteins by red or far-red light promotes the photoconversion to their far-red light–absorbing Pfr state or the red light-absorbing Pr state, respectively. Because prototypical bacteriophytochromes have a parallel dimer architecture, it is generally assumed that symmetric activation with two Pfr state protomers constitutes the signaling-active species. However, the bacteriophytochrome from *Idiomarina* species A28L (*Is*PadC) has recently been reported to enable long-range signal transduction also in asymmetric dimers containing only one Pfr protomer. By combining crystallography, hydrogen–deuterium exchange coupled to MS, and vibrational spectroscopy, we show here that Pfr of *Is*PadC is in equilibrium with an intermediate “Pfr-like” state that combines features of Pfr and Meta-R states observed in other bacteriophytochromes. We also show that structural rearrangements in the N-terminal segment (NTS) can stabilize this Pfr-like state and that the PHY-tongue conformation of *Is*PadC is partially uncoupled from the initial changes in the NTS. This uncoupling enables structural asymmetry of the overall homodimeric assembly and allows signal transduction to the covalently linked physiological diguanylate cyclase output module in which asymmetry might play a role in the enzyme-catalyzed reaction. The functional differences to other phytochrome systems identified here highlight opportunities for using additional red-light sensors in artificial sensor–effector systems.

## Introduction

Light-regulated enzymes gained increased attention over the past decade because of their applicability as optogenetic tools for the precise spatio-temporal control of cellular processes. These modular systems consist of a photosensory module (PSM)^5^ that is functionally linked to a catalytic output module (OM) and thus enables the allosteric regulation of biological responses by light (1). A variety of different PSMs and OMs are available and can be selected specifically to match the requirements of the desired application (2). For optogenetics in mammalian cells, the PSM of bacterial phytochromes (Bphs) has three major advantages over other light-sensing domains: (i) the red-shifted absorption of the biliverdin (BV) cofactor enables light activation in relatively deep layers of biological tissue; (ii) the endogenous production of BV in animal cells; and the (iii) autolyase activity of the apo-phytochrome allow for the assembly of the holoproteins without the requirement for further exogenous maturation enzymes or chromophore addition (2–4). However, to further improve and diversify the applicability of Bph-based optogenetic tools, it is crucial to elucidate how the PSM translates the light signal to the OM.

The PSMs of Bphs are bi-stable photoswitches, which reversibly convert between red-absorbing (Pr) and far-red–absorbing (Pfr) parent states, thereby capable of regulating the dynamic range of a variety of OMs (1). This transition between the PSM states involves a *Z/E* isomerization of the BV chromophore, as well as large-scale changes of the protein backbone (5). Here, the so-called “tongue” motif of the GAF domain switches between an α-helix/coil or a β-sheet/β-hairpin conformation and thus, together with the central helical spine of the dimer, presumably acts as a signal transducer between the chromophore and OM (5–7). Recently, the ∼25-amino acid–long structural element preceding the N-terminal PAS domain (the N-terminal segment, NTS) and providing the cysteine residue involved in covalent attachment of the biliverdin cofactor to the protein backbone has also been shown to tune spectral properties of Bphs and to influence long-range signal transduction (7). Despite the availability of structural and spectroscopic data on the Pr, Pfr, and diverse intermediate states of different Bphs, it is still under debate how chromophore isomerization is linked to the backbone secondary structure transition and ultimately to OM regulation (1). Recently, studies focusing on the naturally-occurring phytochrome-activated diguanylyl cyclase from the bacterium *Idiomarina* sp. A28L (*Is*PadC) have suggested that asymmetric structural rearrangements within the phytochrome dimer eventually regulate OM activity (in this case the OM is a diguanylyl cyclase (DGC)) (8, 9). Furthermore, the structure of an *Is*PadC variant (*Is*PadC^Reg2^) confirmed that a translational motion of one helix in the coiled-coil region connecting the PSM and the DGC domains stabilizes a conformation that stimulates DGC activity (9). This signal transduction mechanism differs from other Bphs and raises the question whether a common mechanism in phytochromes exists or whether there are different communication pathways between the PSMs and OMs, depending on the OM requirements (1).

Based on the crystal structures and UV-visible absorption characteristics, it was proposed that *Is*PadC enables the stabilization of different chromophore environments upon red light illumination to trigger an asymmetric rearrangement of the dark-adapted Pr homodimer. In solution, both BV molecules of the dimer photoisomerize, but only one protomer proceeds to form the Pfr state featuring the conformational refolding of the tongue region, whereas the other protomer arrests in an intermediate state with an isomerized 15*E* chromophore but without full structural rearrangements of the proximal environment, including the tongue region. Previously, this second protomer was tentatively assigned to a Meta-R–like state, and the activated state of IsPadC was therefore termed Meta-R/Pfr heterodimer (9).

Starting from these results (8, 9), we set out to further investigate the activation mechanism with an extended methodic toolbox, including vibrational spectroscopy, which can sensitively access the structural changes of the chromophore and the protein in solution. Although resonance Raman (RR) spectroscopy specifically yields information about the configuration of the BV cofactor, infra-red (IR) difference spectroscopic experiments provide information on the structural changes that occur during the photoconversion of both the chromophore and the protein matrix. Both methods have been widely applied to phytochromes like plant phytochrome A (10–13), the cyanobacterial Cph1 (14, 15), or the bacterial phytochromes *Ag*p1 (16, 17) and *Ag*p2 (6, 18) from the plant pathogen *Agrobacterium fabrum*. Here, the different parent and intermediate states display distinct patterns that are assigned to vibrational normal modes by isotopic labeling experiments (12–14, 19) in combination with the computational normal mode analysis on a quantum mechanics/molecular mechanics (QM/MM) level (19, 20).

In this study, we combined IR and RR spectroscopy with hydrogen-deuterium–exchange MS (HDX-MS) and crystallography of the *Is*PadC chromophore-binding domains in Pr- and Pfr-like states. We show that a Pfr environment can also be stabilized in the absence of the tongue region in Bphs, and we provide evidence for the importance of structural rearrangements in the NTS accompanying Pfr formation. The absence of vibrational signatures for Meta-R–like states in *Is*PadC further highlights the diversity of photoactivation mechanisms in bacteriophytochromes. With respect to long-range signal transduction mechanisms, our results are in line with a structural asymmetry of the tongue region due to partial uncoupling of tongue refolding and Pfr formation in the *Is*PadC system. These functional differences to other phytochrome systems highlight the importance of including different red-light sensors for increasing the chances of obtaining artificial sensor–effector systems suitable for applications in optogenetics.

## Results

### The Illuminated PAS–GAF core forms symmetric homodimers

**Figure 1.**
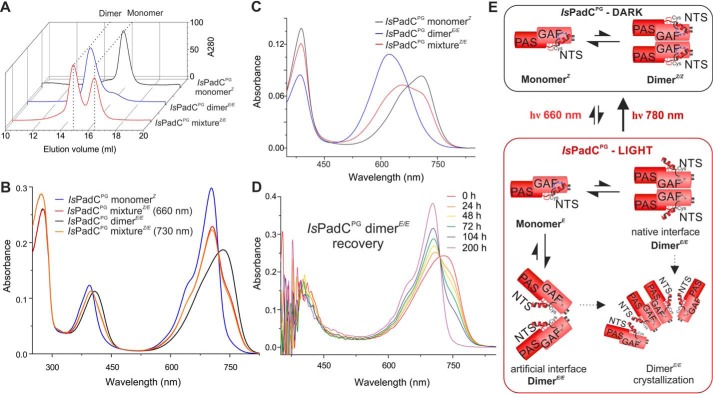
**Biochemical characterization of *Is*PadC^PG^.** *A,* gel-filtration analysis of *Is*PadC^PG^ in the dark and after red light. After illumination, *Is*PadC^PG^ is a mixture of monomers and dimers. *B,* comparison of UV-visible spectra of the *Is*PadC^PG^ monomer*^Z^*, the purified *Is*PadC^PG^ dimer*^E/E^*, and illuminated *Is*PadC^PG^ (*Is*PadC^PG^ mixture*^Z/E^*). Spectra are scaled based on the Soret peak absorption of the illuminated spectra of dimer and monomer species to account for increased purity after the additional purification. *C,* denaturation of *Is*PadC^PG^ in methanol/TCA 0.1%. Illumination of the dimer fraction prior to denaturation confirms the light-induced equilibrium between monomers featuring 15*Z* BV and dimers carrying 15*E* BVs. *D,* thermal recovery of the *Is*PadC^PG^ dimer*^E/E^* in dark conditions at 20 °C and pH 6.0. *E,* schematic representation of *Is*PadC^PG^ oligomerization. In the dark, *Is*PadC^PG^ is purified as a Pr monomer. However, HDX-MS analyses indicate an equilibrium with dimers featuring the native dimer interface (*cf.* Fig. 3*C*). Upon 660 nm illumination, spectral overlap of Pfr-like and Pr *Is*PadC^PG^ populations and potentially higher quantum yields of the Pfr-like to Pr reverse reaction result in a dynamic equilibrium. The Pfr-like monomers can form artificial dimer interfaces stabilizing the Pfr-like environment of each protomer.

To understand which structural elements are responsible for the heterogeneous light-activated state observed in full-length *Is*PadC, we reduced its complexity by generating smaller light-responsive fragments. Because the *Is*PadC photosensory module (*Is*PadC^PSM^, residues 1–500), composed of the PAS, GAF, and PHY domains, features similar spectral properties as full-length *Is*PadC (7), we concluded that the asymmetric behavior of *Is*PadC is an intrinsic property of the PSM. Therefore, we generated the PAS–GAF truncation (*Is*PadC^PG^, residues 1–312) to address the effect of the core chromophore-binding interface on dimer photoactivation. Surprisingly, *Is*PadC^PG^ eluted as a monomeric species from the gel-filtration column when purified under dark conditions. Upon red light illumination, however, a subpopulation of dimers is formed (Fig. 1*A*) that features distinct spectral properties compared with the dark-state monomers (Fig. 1*B*). Acidic denaturation of *Is*PadC^PG^ directly after red-light illumination confirmed the existence of a mixture of 15*Z* and 15*E* BV isomers (Fig. 1*C*), and gel-filtration analysis under constant red-light illumination indicated a rapid equilibrium of dimers and monomers (data not shown) suggesting comparable forward and backward kinetics under 660-nm illumination. The isolated dimeric species, however, was stable if kept in the dark and required days to thermally recover to the Pr state (Fig. 1*D*). The isolation of individual oligomeric species following gel-filtration experiments revealed that the monomer population features a characteristic Pr-state spectrum, whereas the dimer population has a homogeneous UV-visible absorption spectrum resembling a Pfr state even in the absence of the PHY-tongue (Fig. 1*B*). Denaturation of the dimeric species (*Is*PadC^PG^ dimer*^E/E^*) also confirmed the homogeneous BV 15*E* isomer population (Fig. 1*C*). An overview of individual oligomeric states and their correlation with the BV configuration is provided in Fig. 1*E*. To understand the mode of BV stabilization in this dimeric species and to characterize the structural rearrangements defining this Pfr-like state, we crystallized the purified dimeric species, solved its structure, and compared it with the Pr dark-state structure of the purified dark-state monomer species (*Is*PadC^PG^ monomer*^Z^*).

### Crystal structures of the IsPadC^PG^ monomer^Z^ and the IsPadC^PG^ dimer^E/E^ reveal structural elements involved in stabilization of the Pfr-like state

**Figure 2.**
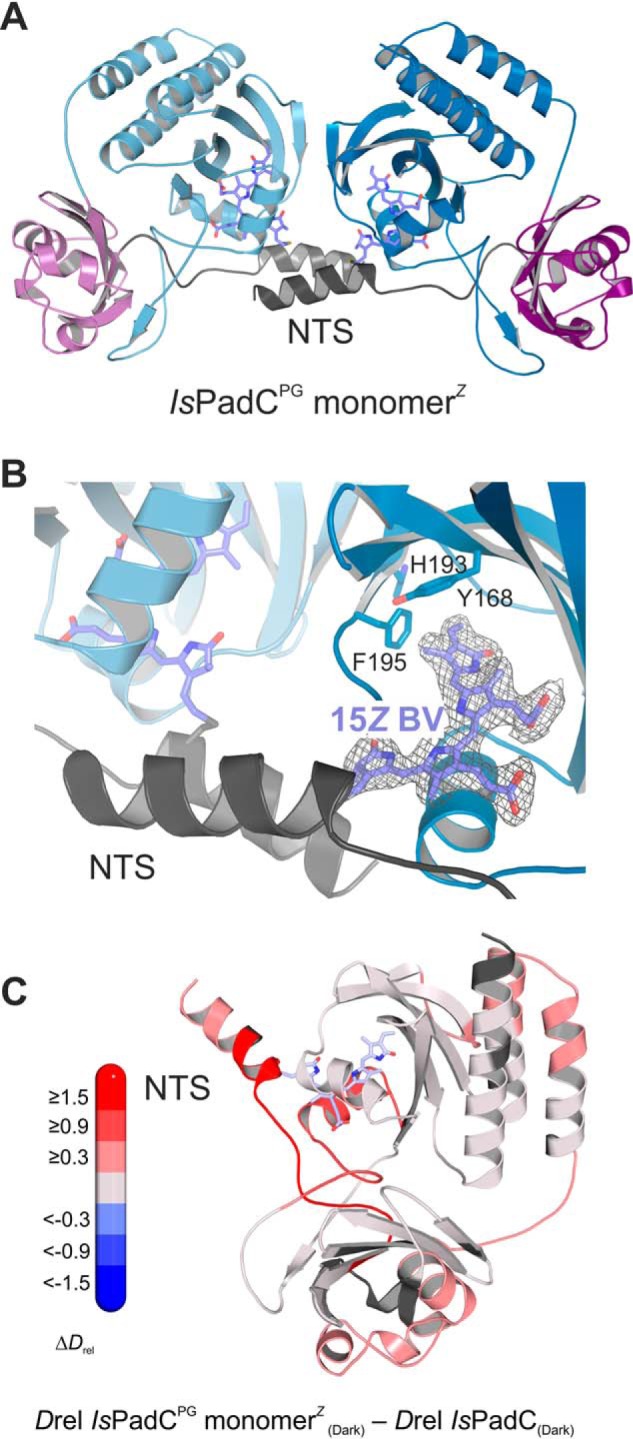
***Is*PadC^PG^ monomer*^Z^* structure and conformational dynamics.** *A,* crystal structure of the *Is*PadC^PG^ monomer*^Z^* showing the two molecules of the asymmetric unit. NTS region, PAS, and GAF domains are colored *gray*, *purple*, and *blue,* respectively. 15*Z* BV is represented as *sticks* and colored *light blue. B*, close-up view of the BV-binding pocket corresponding to the Pr conformation. The 2*F_o_* − *F_c_* electron density map contoured at 1σ around the chromophore is shown as *gray mesh*. Important residues defining the Pr environment of the chromophore are highlighted in *stick* representation. *C,* changes in conformational dynamics upon PAS–GAF truncation of *Is*PadC evaluated by HDX-MS. The structure is colored according to the changes in relative deuterium incorporation (Δ*D*_rel_) between the *IsPadC* dark state and the *Is*PadC^PG^ monomer*^Z^* dark state after 45 s of deuteration. The changes in Δ*D*_rel_ are indicated by the *scale on the left side* with *blue* corresponding to reduced deuterium incorporation and *red* reflecting the increased exchange of amide protons. 15*Z* BV is represented as *sticks* and colored *light blue*.

Crystallization of the *Is*PadC^PG^ monomer*^Z^* under dark conditions revealed the absence of the native *Is*PadC PAS–GAF dimer interface. In line with the gel-filtration experiments, the contacts involved in crystallization (*e.g.* Fig. 2*A*) are most likely not biologically relevant. At 2.4 Å resolution (Table S1), the Pr state for each of the two molecules present in the asymmetric unit was confirmed by the electron density of the chromophore supporting its 15*Z* isomer. Additionally, residues Tyr-168, Phe-195, and His-193 (Fig. 2*B*), which change rotamers upon Pfr formation, are present in identical conformations as observed for the full-length *Is*PadC Pr state structure (8). Similarly, the NTS conformation superposed well with the NTS of the *Is*PadC dark-state structure featuring a β-side linkage of Cys-17 to the chromophore (Fig. 2*B*) (1, 8). Overall, the structure of the *Is*PadC^PG^ monomer*^Z^* is almost identical to the PAS–GAF core of the *Is*PadC Pr structure (RMSD = 0.41 Å over 254 Cα atoms for both chains A and B). The most pronounced changes of both molecules of the asymmetric unit occur in the N-terminal GAF helix (residues 125–135) that is usually part of helix bundles forming the parallel phytochrome dimer interface. Interestingly, analysis of the conformational dynamics of the *Is*PadC^PG^ monomer*^Z^* in solution by HDX-MS showed only a moderate increase in deuterium exchange at the GAF three-helix bundle compared with the full-length protein for longer incubation time points. Although this might be in line with the absence of the native dimer interface in the crystal structure, it also indicates that the extensive interface is most likely still populated in solution but more dynamic than in constructs also featuring the PHY domain (Figs. 1*E* and 2*C*) (8). The HDX-MS analysis further shows that the absence of the PHY-tongue contributes to an increase of conformational freedom in the NTS as well as of the α-helix (residues 239–250) located close to the BV chromophore; both elements constitute a major part of the BV-binding site.

**Figure 3.**
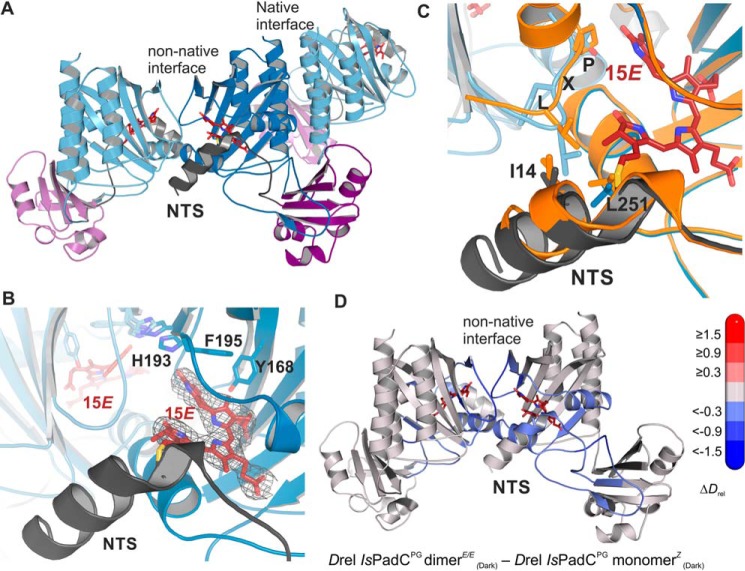
***Is*PadC^PG^ dimer*^E/E^* structure.** *A,* crystallographic model of the *Is*PadC^PG^ dimer*^E/E^*. Three of the eight protomers present in the asymmetric unit are shown to visualize the distinct dimer interfaces observed. NTS region, PAS, and GAF domains are colored *gray, purple,* and *blue,* respectively. 15*E* BV is represented as *sticks* and colored *dark red. B,* close-up view of the Pfr-like BV conformation. The 2*F_o_* − *F_c_* electron density map contoured at 1σ around the chromophore is shown as *gray mesh*. Important residues defining the Pfr environment are shown as *stick* representation. *C,* structural superposition of the *Is*PadC^Reg2^ Pfr state (*orange*, PDB code 6et7 (9)) to the *Is*PadC^PG^ dimer*^E/E^* structure based on the PAS–GAF core (residues 1–312, RMSD = 0.36 Å over 267 Cα atoms). The structural feature indicated with L*X*P corresponds to the start of the L*X*PR*X*SF motif of the PHY-tongue. *L*, Leu-186 in *Is*PadC^PG^ and Leu-446 in *Is*PadC; *X*, Phe-187 in *Is*PadC^PG^ and Gly-465 in *Is*PadC; *P*, Thr-188 in *Is*PadC^PG^ and Pro-466 in *Is*PadC. *D,* changes in conformational dynamics upon *Is*PadC^PG^ dimerization during red light illumination evaluated by HDX-MS. The structure is colored according to the observed changes in relative deuterium incorporation (Δ*D*_rel_) between the *Is*PadC^PG^ dimer*^E/E^* and the *Is*PadC^PG^ monomer*^Z^* dark state after 15 min of deuteration. The changes in Δ*D*_rel_ are indicated by the *scale on the left side* with *blue* corresponding to reduced deuterium incorporation and *red* reflecting increased exchange of amide protons. 15*E* BV is represented as *sticks* and colored *dark red*.

The purified *Is*PadC^PG^ dimer*^E/E^* crystallized only under different conditions, and the elucidation of its structure revealed characteristic differences in the interaction interfaces of individual PAS–GAF molecules (Fig. 3*A*). For each of the eight molecules in the asymmetric unit, the electron density of the BV cofactor was indicative of the 15*E* isomer. Even though the resolution of the corresponding structure is limited to 3 Å, the characteristic sliding of the chromophore in its binding pocket and the altered conformation of residues Tyr-168, Phe-195, and His-193 around the BV (Fig. 3*B*) support the modeling as a 15*E* isomer. In fact, the whole-BV environment resembles that of the Pfr-state protomer of the *Is*PadC^Reg2^ crystal structure (9). Interestingly, also the NTS showed a rearrangement of the α-helix and the architecture of the cofactor linkage, superposing well with the NTS conformation observed in the presence of the PHY-tongue in the Pfr protomer of *Is*PadC^Reg2^ (9). In analogy to Pfr formation in the full-length context, a switch in cofactor coordination by Cys-17 from β- to α-facial is observed after isomerization of the 15*Z* to the 15*E* configuration (Fig. 3*B*).

The eight molecules of the asymmetric unit form two different assembly interfaces. One, defined as the nonnative interface, involves the GAF core region normally covered by the PHY-tongue in full-length *Is*PadC as well as the NTS region, and the second dimer interface (defined as the native interface) involves the GAF three-helix bundles of individual molecules in an identical conformation as observed for longer *Is*PadC constructs (8). A closer look at the nonnative interface revealed that the GAF loop involving residues 184–191 rearranges, allowing Leu-186 to establish hydrophobic contacts with Leu-251 of the GAF core and Ile-14 of the NTS (Fig. 3*C*). HDX-MS analysis of the *Is*PadC^PG^ dimer*^E/E^* further confirmed the formation of this nonnative interface upon *Is*PadC^PG^ monomer*^Z^* illumination by reduced deuterium incorporation in peptides in the rearranged GAF loop and the NTS region (Fig. 3*D* and Fig. S1). Interestingly, Leu-186 superposes well with Leu-464 of the disordered/α-helical PHY-tongue observed for the Pfr-state protomer of *Is*PadC^Reg2^ (9). Moreover, due to the rearrangement of the GAF loop 184–191, the residues 186–188 mimic the conformation of the first part of the L*X*PR*X*SF motif of the PHY-tongue in the Pfr-state. This supports the importance of this motif of the PHY-tongue in bacteriophytochromes for the stabilization of the Pfr-state (1, 21). Interestingly, His-193 shows alternate conformations that correspond to both rotamers previously observed for the *Is*PadC Pr- and Pfr-states (Fig. 3*B*) (8, 9). Most probably, the absence of the α-helical structure of the PHY-tongue in the *Is*PadC^PG^ dimer*^E/E^* enables conformational flexibility of His-193, which might destabilize the nonnative interface and thereby enable the slow recovery of the *Is*PadC^PG^ dimer*^E/E^* and eventual dissociation to the *Is*PadC^PG^ monomer*^Z^*.

Overall, the chromophore environment observed for the *Is*PadC^PG^ dimer*^E/E^* features all the structural characteristics of the Pfr-state observed in *Is*PadC^Reg2^. In the context of the PAS–GAF construct lacking the PHY-tongue element, we define this as a Pfr-like state in the following. To further characterize this Pfr-like state and to address the role of individual structural elements in the overall signal transduction process we performed a vibrational spectroscopy analysis.

### RR spectroscopic analysis of IsPadC and variants

**Figure 4.**
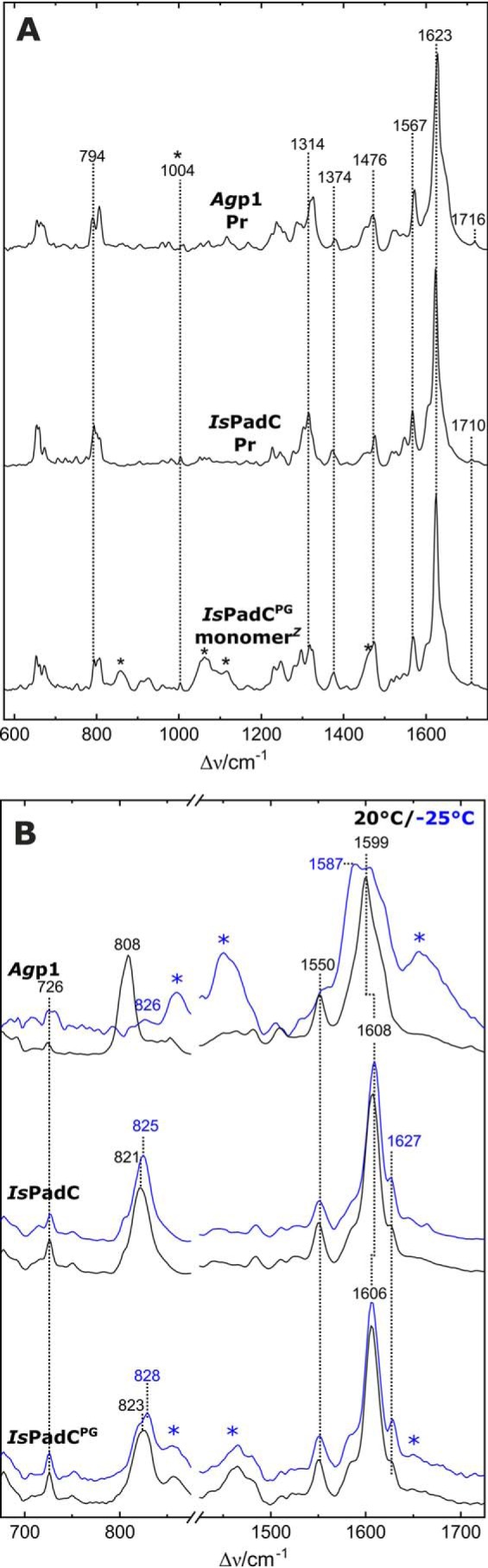
**RR analysis of the various constructs.** *A,* RR spectra in the Pr state. *Top to bottom: Ag*p1, *Is*PadC, and *Is*PadC^PG^ monomer*^Z^*. Contributions from the protein backbone and buffer are marked with *asterisks. B,* RR spectra of photoproduct states obtained by cryo-trapping at different temperatures. The respective samples in the Pr state were illuminated with a 660 nm LED at −25 °C (*blue lines*) and at 20 °C (*black lines*). All RR spectra were measured at −140 °C. Unconverted Pr contributions were subtracted from the spectra. *Top to bottom: Ag*p1, *Is*PadC, and *Is*PadC^PG^. Contributions from the protein backbone and buffer are marked with an *asterisk*.

In the Pr state, *Is*PadC and the *Is*PadC^PG^ monomer*^Z^* exhibit nearly identical RR spectra in the entire spectral range (Fig. 4*A*, Fig. S2, and Table 1). The spectra are also highly similar to the prototypical histidine kinase Bph *Ag*p1, confirming the typical 15*Z* configuration of BV in the Pr state in the truncated construct. *Ag*p1 was chosen as a reference system for this study, because in addition to the large body of spectroscopic (16, 17, 22) and crystallographic data (23), the vibrational mode assignment was recently validated by isotopic labeling experiments complemented by state-of-the-art QM/MM calculations (19). Following this assignment, we attribute the typical N–H in-plane (i.p.) bending mode located at the *B* and *C* rings of BV at 1567 cm^−1^ to a protonation of BV at all four pyrrole rings, resulting in a positively-charged conjugated system.

**Table 1:** **Structural properties defining the state nomenclature**

Protein	Conditions/temperature	Quaternary structure	L*X*P*X*RSF conformation	Secondary structure (PHY-tongue)	Cysteine linkage to chromophore (53)	Chromophore configuration	State nomenclature
Agp1	Dark/20 °C	Dimer	Elongated	β-Hairpin/sheet	β-Side	*ZZZssa*	Pr
	Light/−25 °C	Dimer	N.E.E.*^a^*	β-Hairpin/sheet	α-Side	*ZZEssa*, deprotonated	Meta-Rc
	Light/20 °C	Dimer	α-Helix	Disordered/α-helix	α-Side (N.E.E.)	*ZZEssa*, protonated	Pfr
*Is*PadC PG	Dark/20 °C	Mixture mono/dimer			β-Side	*ZZZssa*	Pr
	Light/−25 °C	Mixture mono/dimer	N.E.E.		α-Side	*ZZEssa*, protonated	Pfr-like
	Light/20 °C	Mixture mono/dimer	L*X*P*X*RSF mimic contact		α-Side	*ZZEssa*, protonated	Pfr-like
*Is*PadC	Dark/20 °C	Dimer	Elongated	β-Hairpin/sheet	β-Side	*ZZZssa*	Pr
	Light/−25 °C	Dimer	N.E.E.	β-Hairpin/sheet	α-Side	*ZZEssa*, protonated	Pfr-like
	Light/20 °C	Dimer	α-Helix or unstructured	Disordered/mixed α-helix and unstructured tongue heterodimers	α-Side	*ZZEssa*, protonated	Pfr-like/Pfr

*^a^* N.E.E. means no experimental evidence.

The RR spectrum of the purified *Is*PadC^PG^ dimer*^E/E^* is identical to the RR spectrum after photoactivation of the monomer*^Z^* sample in the sample compartment of the RR spectrometer (Fig. S3). The only difference is that in this latter case a higher amount of residual Pr contribution needs to be subtracted to account for the 15*Z* BV fraction of the dynamic equilibrium formed under these conditions. The photoactivated spectra of *Is*PadC and *Is*PadC^PG^ both show typical Pfr features such as a dominant C=C stretching peak at 1606 or 1608 cm^−1^, indicative of the 15*E* BV in a Pfr-like state (Fig. 4*B* and Table 1). The characteristic frequency of the B/C N–H i.p. bending mode at 1550 cm^−1^ confirms that BV is also fully protonated in this state. The spectra also display the Pfr-typical intense hydrogen-out-of-plane (HOOP) modes of the *C–D* methine bridge, albeit at somewhat higher frequencies (825 and 828 cm^−1^ in *Is*PadC and *Is*PadC^PG^, respectively) than usually found in the Pfr state of Bphs (∼810 cm^−1^). Because of these similarities, we reason that the Pfr-like state that we described in the crystallographic analysis is also obtained under the experimental conditions of the vibrational spectroscopic experiments.

### RR spectroscopic results: photoactivation experiments at different temperatures

Vibrational spectra of photocycle intermediates, such as Meta-R states, can be obtained either directly through time-resolved pump-probe techniques or alternatively in cryo-trapping experiments (13, 16, 18, 24, 25). In all previous studies, *e.g.* on plant phytochrome A (12, 25, 26) or the Bph *Ag*p1 (16, 17, 27), the Meta-Rc intermediate could be cryo-trapped by illumination with red light (650 nm) in the temperature range between −30 and −25 °C. Because Meta-Rc is the direct precursor of Pfr, we performed photoactivation experiments of both *Is*PadC constructs and *Ag*p1 (full-length protein, including the histidine kinase output module) at different temperatures to selectively populate the Pfr and Meta-Rc states. For RR experiments, samples were illuminated at −25 °C (temperature expected to trap Meta-Rc) and 20 °C (Pfr) and rapidly cooled down to the measurement temperature (−140 °C). The resultant spectra of *Ag*p1 (full-length) are nearly identical to previously published RR spectra of the Meta-Rc and Pfr states of *Ag*p1^PSM^ (28) and similar to other prototypical bacterial phytochromes (Fig. 4*B*) (20). The Pfr state is characterized by a HOOP doublet at ∼808 cm^−1^, an N–H i.p. mode at 1550 cm^−1^, and a main C=C stretching peak at 1599 cm^−1^. Photoactivation at −25 °C results in a typical Meta-Rc spectrum for *Ag*p1, reflected in a broad and poorly-structured envelope, characteristic of a deprotonated (neutral) chromophore (17, 28). The low-resonance enhancement of the chromophore modes of Meta-Rc, which is a consequence of the deprotonation, results in an increase of the relative spectral contribution of the apoprotein such as the amide I band around 1650 cm^−1^ (28). The main C=C stretching feature is downshifted to 1587 cm^−1^, and the shoulder at 1550 cm^−1^ was assigned to a C=C stretching coordinate of the B/C methine bridge. The latter band is largely H/D-insensitive, and no characteristic N–H i.p. band is found in this region, typical of the deprotonated chromophore of Meta-Rc states in canonical phytochromes (28).

RR spectra of the −25 °C photoproducts of *Is*PadC and *Is*PadC^PG^ were recorded under the same conditions as for *Ag*p1. Interestingly, the spectra of both constructs at −25 °C are largely similar to *Is*PadC^PG^ at 20 °C, and only minor differences are found between the two temperatures (Fig. 4*B* and Fig. S3). Specifically, all spectra display a distinct band at 1550 cm^−1^ originating from the N–H i.p. bending mode of the rings *B* and *C*, thus indicating a protonated chromophore in contrast to the Meta-Rc state of *A*gp1 (Fig. 4*B*). Also the well-resolved and narrow bands of the other chromophore modes are consistent with a protonated BV. Moreover, the positions of the HOOP and C=C stretching peaks, which among the various *Is*PadC spectra only differ by ±5 cm^−1^, as well as the N–H i.p. bending mode fall in the range of the corresponding modes of Pfr chromophores, implying that already at −25 °C a Pfr-like chromophore structure is formed. The only minor spectral deviations between *Is*PadC and its truncated construct refer to the frequency of the HOOP mode and indicate slight variations of the dihedral angle of the *C–D* methine bridge (see below) (20).

### IR-difference analysis of IsPadC

**Figure 5.**
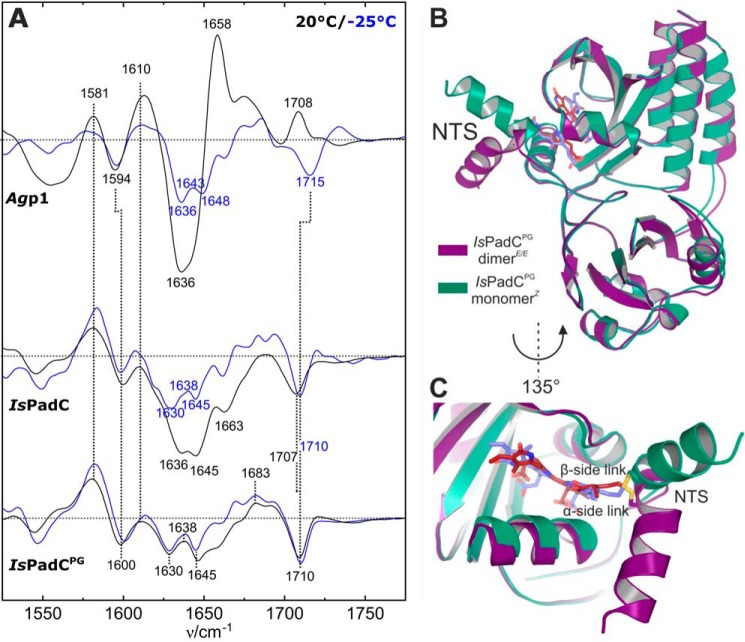
**Photoinduced FTIR difference spectra at different temperatures and structural correlation in *Is*PadC^PG^.** *A,* positive bands represent the respective photoproduct and negative bands the Pr state. Spectra were recorded at *T* = −25 °C (*blue lines*) and *T* = 20 °C (*black lines*). *Top to bottom: Ag*p1, *Is*PadC, and *Is*PadC^PG^. *B* and *C*, structural superposition between chains A of IsPadC^PG^ monomer*^Z^* and IsPadC^PG^ dimer*^E/E^* (RMSD = 0.53 over 257 Cα atoms) (*B*). Close-up view of the structural rearrangement of the NTS and the chromophore linkage to Cys-17 (*C*).

Additional structural information about the chromophore and the protein backbone can be extracted from the light-induced IR difference spectra. Here, negative bands refer to Pr, and positive bands correspond to the photoproduct(s) at the respective temperatures. IR difference spectra were recorded for identical samples at the same temperatures as in the RR experiments to ensure maximum comparability of the two datasets. However, although the RR measurement temperature was −140 °C, the IR spectra were accumulated during the illumination period at temperatures of 20 or −25 °C (Fig. 5). Because these deviations in the measurement temperature are necessary to avoid artifacts in both types of experiments, it should be noted that they could lead to minor temperature-dependent differences in band positions.

**Figure 6.**
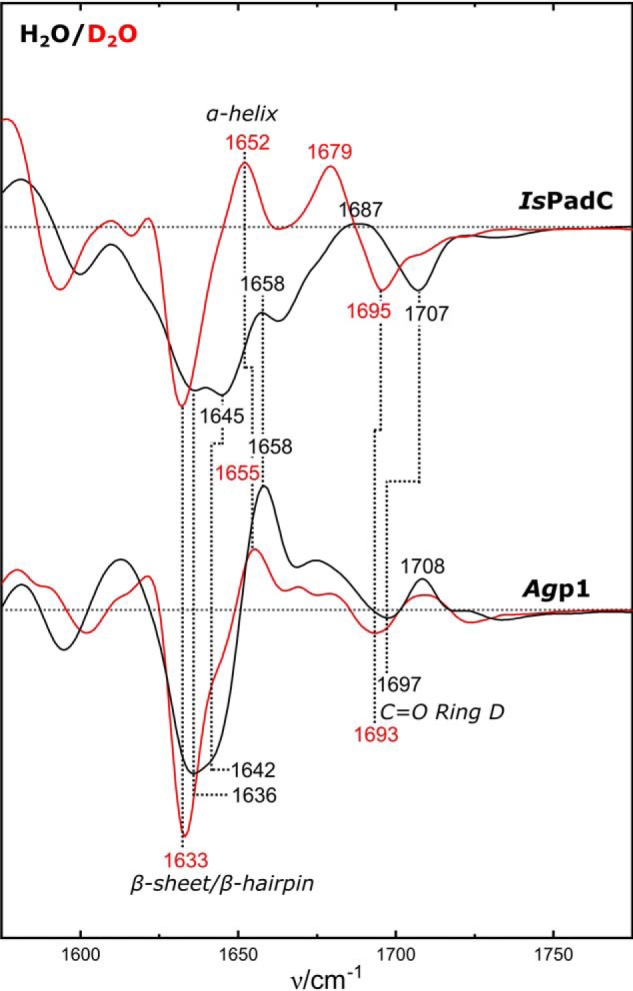
**D_2_O effect on FTIR difference spectra.** Pfr-minus-Pr difference IR spectra of IsPadC and Agp1 at 20 °C in H_2_O buffer (*black lines*) and D_2_O buffer (*red lines*) buffer. Positive bands originate from the Pfr state and negative bands from the Pr state.

In previous experiments with *Ag*p1^PSM^, photoactivation at −30 °C and rapid-scan IR experiments at 25 °C with a delay time of 20 ms yielded identical IR-difference signatures that were assigned to the formation of the Meta-Rc state (16), consistent with the RR experiments (28). These results validated the cryo-trapping method as an alternative to rapid-scan experiments and further demonstrated that spectral changes due to different measurement temperatures are negligible. The present results for full-length *Ag*p1 at −25 °C and 20 °C resemble those for *Ag*p1^PSM^ (Fig. 5 and Table 1). For both temperatures, the distinct marker bands that are assigned to the C=O groups of the chromophore (>1680 cm^−1^), the amide I modes of the protein backbone (1620–1680 cm^−1^), and the C=C stretching of the chromophores B/C methine bridge (1581(+)/1594(−) cm^−1^) were reproduced under the present conditions. Furthermore, the characteristic downshifts of all marker bands, *e.g.* the amide I (1633 cm^−1^) and ring *D* C=O stretching mode (1693 cm^−1^), are observed after D_2_O buffer exchange, confirming the established *Ag*p1^PSM^ band assignment (16) for the full-length construct (Fig. 6).

As in the RR experiments, the spectral signatures associated with the chromophore reactions of *Is*PadC and PG are largely similar at −25 °C and 20 °C but differ from those of *Ag*p1. In the C=O stretching region, a dominant-negative band at 1707 cm^−1^ is observed in both variants at 20 °C (Fig. 5) The small upshift to 1710 cm^−1^ at −25 °C can be ascribed to a temperature effect. In *Is*PadC, H/D exchange causes a downshift of this band to 1695 cm^−1^ (Fig. 6), coincident with the position of the ring *D* C=O stretching band of *Ag*p1 in D_2_O buffer. Both *Is*PadC variants exhibit the characteristic *B/C* C=C stretching band pair at (1581(+)/1600(−) cm^−1^), reflecting the *Z*/*E* isomerization of BV.

The most prominent differences between the two measurement temperatures were observed in the amide I region. At −25 °C, a band pattern (1630(−), 1638(+), and 1645(−) cm^−1^) with low intensity is observed in *Is*PadC, closely resembling the spectrum of *Ag*p1 at the same temperature, albeit with small shifts in the band positions (1636(−), 1643(+), and 1648(−) cm^−1^) (Fig. 5*A*). At 20 °C, the difference spectrum of *Is*PadC^PG^ remains unchanged compared with −25 °C except for small variations in the baseline. In contrast, *Is*PadC exhibits additional negative signals at 1636 and 1645 cm^−1^ of substantial intensity. These increased amide I difference signals indicate that secondary structure changes are associated with the phototransformation of full-length *Is*PadC at 20 °C. The corresponding difference spectrum of *Ag*p1 also displays a strong negative band at ∼1636 cm^−1^, which is accompanied by a prominent positive peak at 1658 cm^−1^. Such a signal pair has been observed in the “Pfr-minus-Pr” IR difference spectra of various prototypical phytochromes, including *Rp*BphP2 from *Rhodopseudomonas palustris* (29), *Ag*p1 (16), and *Dr*BphP (30), cyanobacterial Cph1 (14), as well as the full-length phyA and its photosensor domain (12, 13, 31). These signals were attributed to the structural transition of the tongue in the PHY domain from β-sheet/β-hairpin in Pr (negative 1636 cm^−1^ peak) to an α-helix/disordered region in Pfr (positive 1658 cm^−1^ peak). However, the relative amplitudes and their positions vary considerably, although they refer to the same or very similar structural changes. These variations originate from an intrinsic limitation of IR difference spectroscopy if the conjugate absolute bands that undergo a reaction-induced change exhibit large band widths, similar peak frequencies, and different absorption cross-sections as in the case of the amide I bands. Then the positive and negative signals may overlap leading to a partial cancellation of the peaks. This seems to be the case for *Is*PadC, which shows a broad negative difference signal with peaks at 1636/1645(1647) cm^−1^, but it lacks the positive counterpart at ∼1658 cm^−1^ (Fig. 5). However, the amide I band changes can also be identified in the spectra measured after H/D exchange, which causes a downshift of the amide I bands and affects band widths and absorption cross-sections, thereby altering the complex pattern of difference signals (Fig. 6). Interestingly, for *A*gp1 the peak cancellation is more severe in D_2_O than in H_2_O such that the resultant (positive) 1655 cm^−1^ band is distinctly weaker than its counterpart in H_2_O (1658 cm^−1^). However, the negative (1633 cm^−1^) and positive signals (1652–1655 cm^−1^) are now readily detectable for *Is*PadC. Thus, the IR spectroscopic findings for *Is*PadC are fully compatible with the secondary structure transition from β-sheet/β-hairpin to α-helix during the Pr → Pfr phototransformation at 20 °C in at least one protomer (9). In contrast, this conformational transition is largely blocked at −25 °C in *A*gp1 and *Is*PadC and, due to the lack of the PHY domain, cannot occur in *Is*PadC^PG^ at all.

As expected, the structural changes of the tongue are fully reversible in *Ag*p1 and *IsPadC* at ambient temperature as shown by the IR difference spectra obtained for the forward and backward photoconversion using 660 and 750 nm irradiation, respectively (Fig. S4, gray traces). However, also the spectral changes induced by irradiation at −25 °C can be largely reverted at the same temperature in the case of *Is*PadC^PG^, albeit not for *Is*PadC and *Ag*p1.

## Discussion

### Tongue refolding is preceded by structural rearrangements in the NTS

The comparative analysis of the IR difference spectra of *Ag*p1 and *Is*PadC revealed large-scale changes of the protein in the last step of the Pr → Pfr photoconversion. This is readily attributed to the secondary structure conversion of the tongue segment of the PHY domain because the corresponding IR difference signals were not observed for *Is*PadC^PG^. However, the *Is*PadC^PG^ construct also displays difference signals in the amide I region, although the amplitudes are relatively low (Fig. 5*A*). These spectral changes are likely to reflect only minor perturbations of the protein structure and must originate from the PAS–GAF domains. The IR difference spectra of *Is*PadC^PG^ are very similar, albeit not identical, to those of *Is*PadC measured at −25 °C. These findings imply that the phototransformation of Pr prior to tongue refolding involves structural changes associated with the PAS–GAF domains, independent of the presence or absence of the PHY domain. The low signal amplitudes rule out a genuine secondary structure change but may reflect altered intramolecular contacts of individual peptide segments. These structural changes in the PAS–GAF domains can be rationalized on the basis of the present crystallographic analyses of the *Is*PadC^PG^ monomer*^Z^* and dimer*^E/E^* that reveal a reorientation of the NTS helix and the switch from the β-facial to α-facial coordination of the BV cofactor during the Pr → Pfr conversion (Fig. 5*B*). Evidently, these structural changes occur prior to the restructuring of the tongue in the PHY domain as shown by the temperature-dependent IR difference spectra of *Is*PadC and *A*gp1. This sequence of structural rearrangements, starting in the PAS–GAF domain and followed by the tongue in the PHY domain, is presumably not unique for *Is*PadC but may represent a common mechanism in phytochromes. For the engineered variant PAiRFP2 of the bathy phytochrome *A*gp2 the crystallographic analysis also demonstrated the transition from α- to β-facial chromophore coordination in the Meta-F intermediate of the Pfr → Pr phototransformation (6).

### Chromophore structural changes

Although the IR difference spectra of *Is*PadC and *Ag*p1 are very similar at −25 °C and indicate the lack of the tongue restructuring, the chromophore structures are quite different as revealed by the RR spectra. In *Ag*p1, the Meta-Rc state, which is stabilized at this temperature, shows a deprotonated BV in which a proton has been dissociated either from ring *B* or *C* pyrrole nitrogen (17, 27, 28). A Meta-Rc intermediate with a deprotonated chromophore was also found in other BV-binding prototypical phytochromes such as *Dr*BphP from *Deinococcus radiodurans* (32). In contrast, the RR spectra of both *Is*PadC constructs measured at −25 °C clearly demonstrate a protonated chromophore. Moreover, the RR spectra resemble the final product of the Pr phototransformation, *i.e.* the Pfr state, obtained at 20 °C. Thus, for the sake of simplicity, we refer to this intermediate of *Is*PadC as “Pfr-like” rather than “Meta-R-like” as suggested previously (9).

Stabilization of a Pfr-like protonated chromophore in the absence of the PHY domain is in contrast to the behavior of other BV-binding prototypical phytochromes such as *Dr*BphP (32) or *Ag*p1 (33), which afforded a final photoproduct with a deprotonated chromophore closely related to that of the Meta-Rc states of the respective complete photosensor modules. However, there is a good agreement with the results obtained for the PG truncation variants of BV-binding prototypical *Rp*BphP2 (34) and bathy *Rp*BphP6 from *R. palustris* (35). In both cases, the RR spectra display Pfr-like vibrational signatures of a protonated *ZZEssa* configuration with unusually high HOOP frequencies. Apparently, there are system-specific differences in the photocycles of members of the phytochrome superfamily, frequently brought about by a protonation-dependent heterogeneity of different parent or intermediate states, in line with previous results (36–40). Eventually, this heterogeneity influences structural changes involved in signal propagation (41) and, together with other properties of the bilin cofactor, might be influenced by the degree of structural coupling with functional elements affecting the BV environment (7, 42) and hence the stability of specific cofactor configurations, conformations, and/or protonation states.

### Coupling of chromophore and protein structural changes

The failure to detect a transient chromophore de- and reprotonation in the various *Is*PadC constructs might, on the first sight, point to a reaction mechanism of the Pr phototransformation, different from those of other prototypical phytochromes. However, we cannot rule out *per se* the existence of an intermediate with a deprotonated chromophore.

Instead, we propose an alternative interpretation that takes into account the boundary conditions for detecting intermediates. Intermediate states can only be detected in time-resolved experiments at ambient temperature if their transient accumulation is sufficiently high. This prerequisite corresponds to activation barriers that are lower for the formation than for the decay. Hence, intermediates can only be trapped cryogenically if the energy barrier for the respective decay reaction is sufficiently high at the trapping temperature. In more general terms, the successful detection of intermediates crucially depends on the energy landscape along the reaction pathway. Thus, the proposed alternative interpretation assumes that the Pr → Pfr transformation may follow a common *general* mechanism running via similar intermediate states that, however, cannot be trapped in each case due different protein-specific energy barriers.

The general mechanism may then be summarized as follows. The transition to the Meta-Rc state is associated with (i) the conversion of the β- to α-facial chromophore binding configuration, (ii) the reorientation of the NTS helix, (iii) the deprotonation of the tetrapyrrole, and (iv) the proton translocation to bulk solution. The subsequent proton re-uptake and chromophore re-protonation in the Pfr-like precursor is then followed by the secondary structure transition of the tongue segment. The final step to Pfr involves adjustments of the chromophore geometry. Either the Meta-Rc or the Pfr-like state (or both) constitutes a stage of bifurcation. Previous time-resolved RR spectroscopic studies have demonstrated a thermal short-cut reaction back to the Pr from the Meta-state, in competition to its decay route to Pfr (22). Clearly, this proposed general reaction mechanism must currently be considered as a hypothesis that deserves further experiments for its verification or refinement.

### Impact of the quaternary structure on the chromophore conformation

Despite the overall striking similarities of the RR spectra of the photoproducts of *Is*PadC^PG^ and *Is*PadC, small but clearly detectable differences are noteworthy. These spectral differences refer to modes localized at the *C–D* methine bridge. In both protein variants, the frequency of the *C–D* HOOP mode decreases by 4–5 cm^−1^ upon going from the photoproduct at −25 to that at +20 °C. Furthermore, at both temperatures the frequency difference between the photoproducts of *Is*PadC^PG^ and *Is*PadC is 2–3 cm^−1^ and thus similar to the frequency difference of the temperature-independent *C–D* stretching mode. Based on a previous vibrational analysis of the Pfr chromophore of Agp2, these small frequency shifts correspond to slight differences in the *C–D* dihedral angles (20) and indicate that the *C–D* methine bridge most sensitively responds to differences in the interactions with the protein environment. The temperature-independent spectral differences between the respective photoproducts of *Is*PadC^PG^ and *Is*PadC are readily attributed to the absence or presence of the PHY domain, respectively, and demonstrate its conformational coupling with the chromophore-binding site, eventually influenced by the structure of the tongue element. The temperature-dependent spectral changes between the photoproducts at −25 and +20 °C are consequently attributed to the restructuring of the PHY-tongue segment in the last step of the photoconversion of the full-length protein. Because these changes are very similar in *Is*PadC^PG^ and *Is*PadC, the tongue-mimic of the artificial dimer interface in *Is*PadC^PG^ apparently helps to stabilize the Pfr-like conformation in the absence of the tongue element.

This interpretation is in line with previous findings on *Dr*BphP, which suggested that the tongue transition constitutes a thermal equilibrium that is affected by but not strictly linked to the chromophore state (42). The degree of tongue coupling with the cofactor apparently not only influences the thermal back reaction to Pr (see below) but also the efficiency of the photochemical reaction. Because of spectral overlap of Pr and Pfr states in the region between 630 and 730 nm, activation with red light frequently results in steady-state populations of predominantly Pfr but partial Pr contributions. The α-helical tongue conformation generally stabilizes Pfr (1, 21) by reducing the quantum yield of the Pfr to Pr light reaction. The absence of the tongue in *Is*PadC^PG^ results in a rapid equilibrium of Pfr-like and Pr states, which can only partially be compensated by the short tongue mimic of one GAF loop in the nonnative dimer interface. Consequently, also amino acid substitutions in the tongue region (32, 43) and the NTS (7) frequently have a profound impact on spectral properties of the light-activated state and its recovery to the ground state. The weaker coupling of the PHY-tongue with the BV cofactor observed in *Is*PadC could therefore account for higher quantum yields of the light reaction from Pfr to Pr and in part explain the characteristic UV-visible absorption spectra during red-light illumination as a mixture of Pfr, Pfr-like, and Pr states.

The relatively strong protein–protein interactions and their impact on the chromophore structure in the individual protomers of parallel phytochrome dimers may be the basis for the uncoupling mechanism of *Is*PadC that allows the system to form Pfr *and* Pfr-like states upon illumination. The recently proposed heterodimer model for long-range signal transduction in *Is*PadC (9) hence could be realized by the combination of a Pfr state protomer with an α-helical tongue element and the second protomer in a Pfr-like state with a tongue region that is not (fully) restructured. This interpretation can well-account for the multiexponential thermal back reaction (9), because it involves chromophore isomerization *and* tongue refolding for the Pfr state, whereas it is largely restricted to the chromophore isomerization in the Pfr-like state. In addition, structural heterogeneity of the tongue region was previously also observed in bimodal exchange characteristics of the HDX-MS data (8). Complementing insights from the IR measurements, HDX data enable the direct characterization of structurally different conformations within the ensemble mixture provided that their exchange kinetics are significantly faster than the interconversion between the two conformations. Because no bimodal exchange characteristics could be observed in the cofactor environment of the GAF domain and the NTS region, these data further support the weak coupling of cofactor structure with the tongue conformation in *Is*PadC and that the structural changes in the NTS occur prior to and independent of the tongue rearrangements.

### Summary and implications

To understand the structural prerequisites for Pfr-state formation in *Is*PadC, we combined vibrational spectroscopy analyses with crystallographic observations in full-length and chromophore-binding core constructs of *Is*PadC. The comparison of the *Is*PadC^PG^ structure in dark and light states showed that the 15*E* configuration of the chromophore in a Pfr-like environment can already be stabilized in constructs lacking the PHY-tongue region. RR analysis indicates very similar 15*E* protonated BV environments in both the full-length *Is*PadC and in the *Is*PadC^PG^ dimer*^E/E^* that lacks a disordered/α-helical PHY-tongue. Thus, these findings show that the observed structural rearrangements in the NTS precede tongue refolding and, thereby, are sufficient to allow the stabilization of a Pfr-like state in full-length *Is*PadC. This implies that dimers featuring one Pfr-like state and one Pfr state, but also combinations of one Pr protomer with a Pfr protomer, could provide the structural asymmetry of the phytochrome-sensory module that is sufficient for activation of the diguanylate cyclase output in solution (9). Considering the relevance of asymmetric intermediates as part of the GGDEF mechanism for formation of the symmetric final product cyclic dimeric-GMP (44), the observed asymmetry in all PadC members characterized so far (8) might play an important role for the efficient stimulation of the corresponding diguanylate cyclase activity. Although the formation of the bacterial second messenger cyclic dimeric-GMP, which is involved in the regulation of lifestyle decisions (44), might benefit from such an asymmetric molecular mechanism, other bacteriophytochrome-linked effector domains might not require this type of structural asymmetry. With a variety of biological functionalities being regulated by phytochrome sensors, ranging from histidine kinases to phosphatases or protein–protein interaction modules (1), it is not surprising that nature has used a broad range of strategies for regulating diverse effector domains by phytochromes. Although symmetric activation appears to be relevant for some, different degrees of asymmetry are functionally relevant for others and might also be used by evolution to tune the dynamic range of the regulated functionality (1). The importance of asymmetry for allosteric regulation is clearly not restricted to phytochromes or other dimeric photoreceptors, but it has recently also been established for protein families such as G-protein–coupled receptors (45), RAF kinases (46), and nucleotide-binding sensors (47). Unraveling details of the molecular mechanisms involved in defining asymmetric properties consequently contributes to a better appreciation of an important tuning mechanism employed by nature. Eventually, such mechanisms could be applied for rational engineering approaches, for example, rational designs of optogenetic tools employing photoreceptor modules with specific asymmetric properties.

## Experimental procedures

### Protein expression and purification

The PAS–GAF truncation (*Is*PadC^PG1–312^) was generated from pET M11 *Is*PadC (8) by site-directed mutagenesis following the protocol described by Liu and Naismith (48) using the primer pairs listed in Table S2.

The His_6_-tagged holoproteins were expressed in BL21 (DE3) containing the previously generated pT7-ho1 helper plasmid (8) to co-express heme oxygenase (HO-1) allowing *in vivo* synthesis of biliverdin as described previously (8, 9). Holoproteins were purified, as described in detail previously (9), and stored at −80 °C in storage buffer (10 mm Hepes, pH 7.0, 500 mm NaCl, 2 mm MgCl_2_).

To specifically isolate the activated dimer fraction of *Is*PadC^PG1–312^, the purified protein was illuminated (660 nm, 45 milliwatts cm^−2^) for 2 min at room temperature prior to loading onto a 10/300 Superdex 200 increase analytical grade column at 4 °C under nonactinic light conditions. Because red-light illumination produced a dynamic equilibrium of monomer and dimer conformations, the purification procedure was repeated several times to enrich the overall dimer fraction.

Agp1 samples were prepared according to published protocols in a Tris buffer containing 50 mm Tris-Cl, 5 mm EDTA, and 300 mm NaCl and adjusted with HCl and NaOH to pH 7.8 (49). In D_2_O experiments, the corresponding pD = 7.8 was adjusted with DCl according to an apparent pH of 7.4

### Protein crystallization

The *Is*PadC^PG^ monomer*^Z^* and *Is*PadC^PG^ dimer*^E/E^* were crystallized at 293 K under dark conditions using a sitting-drop vapor-diffusion setup. For the *Is*PadC^PG^ monomer*^Z^*, the best diffracting crystals were obtained in 0.6-μl drops containing equal volumes of protein solution (10 mg ml^−1^), and reservoir solution (0.24 m sodium malonate, pH 7.0, 20% PEG 3350) equilibrated against 35 μl of reservoir solution. Crystals appeared after overnight incubation and reached final dimensions within 5 days. For the *Is*PadC^PG^ dimer*^E/E^*, the best diffracting crystals were obtained in 0.6-μl drops containing 0.2 μl of protein solution (10 mg ml^−1^), 0.1 μl of crystal seeds from the initial *Is*PadC^PG^ dimer*^E/E^* crystals obtained in another condition (after mixing equal volumes of protein solution at 10 mg ml^−1^ with 0.1 m sodium malonate, pH 5.0, 12% PEG 3350), and 0.3 μl of reservoir solution (0.1 m BisTris, pH 6.5, 20% PEG 5000 monomethyl ether) equilibrated against 35 μl of reservoir solution. Crystals appeared after overnight incubation and reached the final dimension after 48 h.

Crystals were harvested under low-intensity nonactinic green-light conditions (520 ± 20 nm LED) by transferring the crystals to a cryoprotectant solution (reservoir solution containing 10% glycerol) and subsequent flash-freezing in liquid nitrogen. Diffraction data for *Is*PadC^PG^ monomer*^Z^* were collected at beamline ID23-2 of the European Synchrotron Radiation Facility (ESRF), and *Is*PadC^PG^ dimer*^E/E^* diffraction data were collected at beamline P11 of the Deutsches Elektronen-Synchrotron (DESY). Data were processed using the XDS program package (50) (Table S1).

The crystal structure of the *Is*PadC^PG^ monomer*^Z^* was solved by molecular replacement using PHENIX Phaser (51) with one protomer of the PAS–GAF fragment of the full-length *Is*PadC (PDB 5llw). Two molecules of PAS–GAF were successfully placed in the asymmetric unit. The crystal structure of the *Is*PadC^PG^ dimer*^E/E^* was solved by molecular replacement using PHENIX Phaser with the PAS–GAF fragment of the Pfr protomer of *Is*PadC^Reg2^ (PDB 6et7). Eight molecules of PAS–GAF were successfully placed in the asymmetric unit. Although molecular replacement with a Pr protomer in analogy to the dark-state monomer was also successful, the difference densities observed in the initial model clearly indicated the 15*E* configuration of BV and the Pfr state rotamers of critical residues in its surroundings. The initial models obtained were then manually adapted for the regions that showed some deviation from the search models in several rounds of maximum-likelihood refinement of the models modified with Coot using α_A_-weighted 2m*F_o_* − D*F_c_* and *F_o_* − *F_c_* electron density maps. In addition, torsion-NCS restraints and secondary structure restraints were included. During the final rounds of refinement, optimization of X-ray and ADP weights was performed.

### UV-visible characterization

UV-visible absorption spectra were acquired with a CCD-based Specord S300 UV-visible spectrophotometer using samples equilibrated at 20 °C and diluted in storage buffer. Dark-adapted absorption spectra were measured under nonactinic conditions by minimizing the contact time with measuring light using a neutral density filter (ND = 2.0) between the light source and the sample cuvette. Illuminated spectra were recorded under constant red-light irradiation (660 nm, 45 milliwatts cm^−2^) in the presence of the same neutral density filter to attenuate the measuring light. To record the UV-visible absorption spectra of the denatured biliverdin-bound proteins, we followed our previously described method (9) adapted from Thümmler *et al.* (52).

### IR difference spectroscopy

IR spectroscopic measurements were carried out in the transmission mode using a Bruker FTIR spectrometer (Tensor27), equipped with liquid nitrogen-cooled mercury-cadmium-telluride (MCT) detector. IR single channel (SC) spectra were calculated by Fourier transformation of the average from 200 single interferometer scans (∼4-min measurement time) in the range between 1100 and 1800 cm^−1^. IR difference (IR_diff_) spectra were subsequently calculated directly from the IR intensity on the detector (SC) recorded before and after illumination, using a 660-nm LED array (IR_diff_ = −log (SC_illu_/SC_dark_)), which equals the difference of the respective absorbance spectra. For photochemical back-conversion experiments, a similar LED array (750 nm) was used. The LED array consisted of 12 single LEDs each with a power of 30 milliwatts that were placed on both sides of the IR cell to ensure uniform illumination of the sample. The sample temperature was controlled with a home-built liquid N2-cooled bath cryostat. Baseline drifts were corrected with a polynomial baseline correction with the OPUS software package.

### RR spectroscopy

RR measurements were performed using a Bruker Fourier-transform Raman spectrometer (RFS 100/S) with 1064-nm excitation (Nd-YAG cw laser, line width 1 cm^−1^), equipped with a nitrogen-cooled cryostat from Resultec (Linkam). All spectra of the samples in frozen solution were recorded at approximately −140 °C with a laser power at the sample of 690 milliwatts and accumulation time of 1 h (2000 scans). To identify potential laser-induced damage of the phytochrome samples, RR spectra before and after a series of measurements were compared. In no case, were changes between these control spectra determined. For photochemical conversion experiments at the respective temperatures, the samples were illuminated with a 660-nm LED, and the illumination protocol was adapted from previous works (25, 28). Subtraction of residual Pr contributions and a polynomial baseline correction were performed with the OPUS software package.

## Author contributions

D. B., G. G., P. H., and A. W. conceptualization; D. B., G. G., P. H., and A. W. data curation; D. B. and G. G. formal analysis; D. B., G. G., and N. M. investigation; D. B. and G. G. visualization; D. B., G. G., M. M., P. H., and A. W. methodology; D. B. and G. G. writing-original draft; D. B., G. G., P. H., and A. W. project administration; D. B., G. G., P. H., and A. W. writing-review and editing; M. M., P. H., and A. W. resources; P. H. and A. W. supervision; P. H. and A. W. funding acquisition; P. H. and A. W. validation.

## Supplementary Material

Supporting Information

## Footnotes

5 The abbreviations used are:

PSM: photosensory module
OM: output module
GAF: cGMP-specific phosphodiesterases-adenylyl cyclases and FhlA
PAS: Per-ARNT-Sim
NTS: N-terminal segment
RMSD: root mean square deviation
Bph: bacterial phytochrome
BV: biliverdin
DGC: diguanylyl cyclase
HDX-MS: hydrogen-deuterium–exchange MS
QM/MM: quantum mechanics/molecular mechanics
PDB: Protein Data Bank
HOOP: hydrogen-out-of-plane
BisTris: 2-[bis(2-hydroxyethyl)amino]-2-(hydroxymethyl)propane-1,3-diol
RR: resonance Raman
SC: single channel
i.p.: in-plane
H/D: hydrogen/deuterium.
