# Supplementary material for: Distinct chromophore–protein environments enable asymmetric activation of a bacteriophytochrome-activated diguanylate cyclase
Source: J Biol Chem. 2019 Dec 4;295(2):539–51. doi: 10.1074/jbc.RA119.011915 (PMC6956517; doi:10.1074/jbc.RA119.011915)
Supplement: Supporting Information [file supp_295_2_539__index.html]

Distinct chromophore-protein environments enable asymmetric activation of a bacteriophytochrome activated diguanylate cyclase — Structural asymmetry of a phytochrome — Distinct chromophore–protein environments enable asymmetric activation of a bacteriophytochrome-activated diguanylate cyclase — Structural asymmetry of a phytochrome — Supporting Information 

# Distinct chromophore–protein environments enable asymmetric activation of a bacteriophytochrome-activated diguanylate cyclase

## Supporting Information

- Supporting Information (to be published online) - Supplementary Tables 1 to 2 Supplementary Figures 1 to 4
