# Supplementary material for: Distinct chromophore–protein environments enable asymmetric activation of a bacteriophytochrome-activated diguanylate cyclase
Source: J Biol Chem. 2019 Dec 4;295(2):539–51. doi: 10.1074/jbc.RA119.011915 (PMC6956517; doi:10.1074/jbc.RA119.011915)
Supplement: Supporting Information [file supp_RA119.011915_157149_1_supp_438465_q1zbrj.pdf]

Supporting Information for

Distinct chromophore-protein environments enable asymmetric activation of a bacteriophytochrome-activated diguanylate cyclase.

David Buhrke[1]†*, Geoffrey Gourinchas[2]†, Melanie Müller[3], Norbert Michael[1],

Peter Hildebrandt[1] and Andreas Winkler[2]*

*For correspondence: (andreas.winkler@tugraz.at), (david.buhrke@campus.tu-berlin.de)

This PDF file includes:

Supplementary Tables 1 to 2

Supplementary Figures 1 to 4

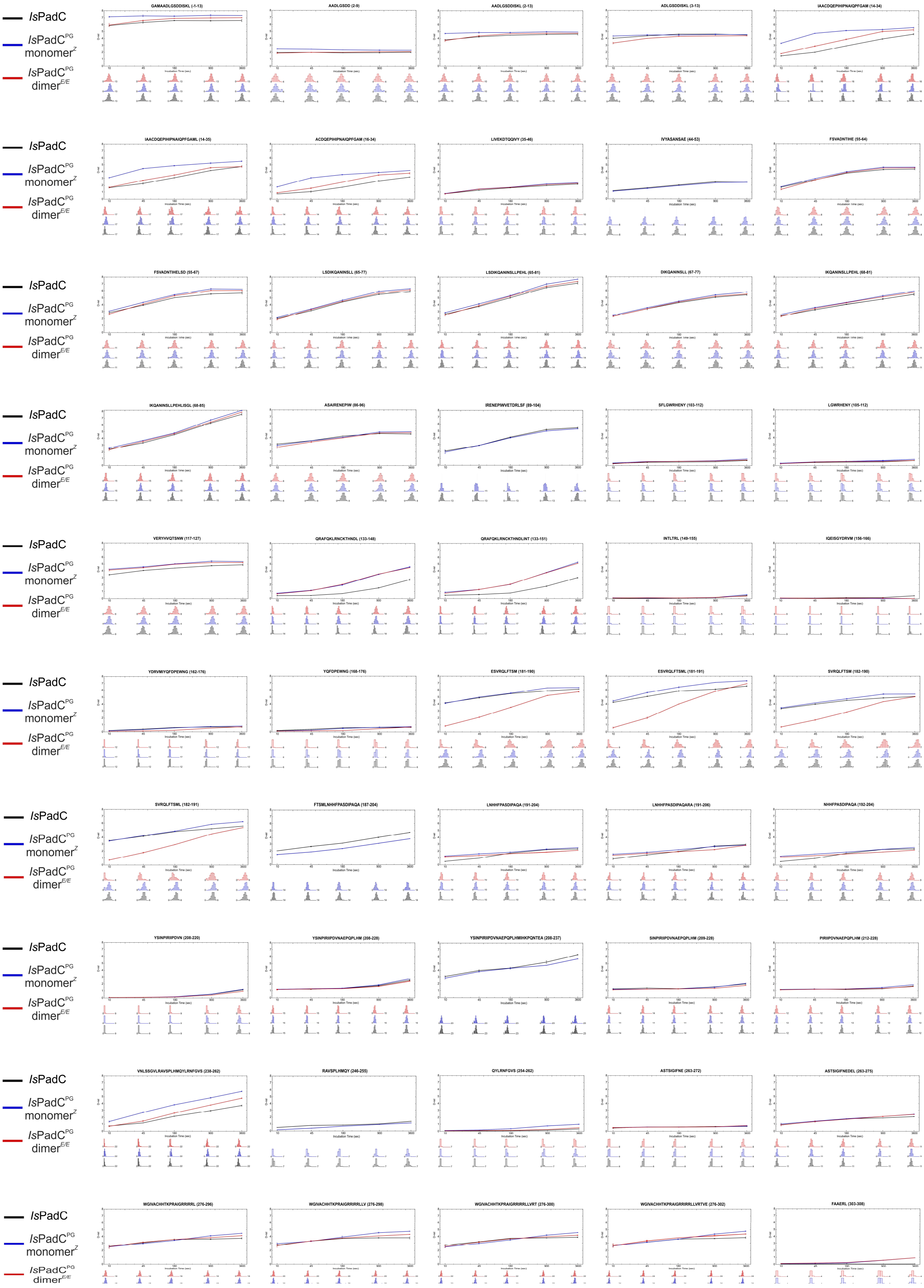
IsPadC
IsPadC^PG monomer^Z
IsPadC^PG dimer^E/E
GAMAADLGSDDISKL (-1-13)
AADLGSDD (2-9)
AADLGSDDISKL (2-13)
ADLGSDDISKL (3-13)
IAACDQEPIHIPNAIQPFGAM (14-34)
IAACDQEPIHIPNAIQPFGAML (14-35)
ACDQEPIHIPNAIQPFGAM (16-34)
LIVEKDTQQIVY (35-46)
IVYASANSAE (44-53)
FSVADNTIHE (55-64)
FSVADNTIHELSD (55-67)
LSDIKQANINSLL (65-77)
LSDIKQANINSLLPEHL (65-81)
DIKQANINSLL (67-77)
IKQANINSLLPEHL (68-81)
IKQANINSLLPEHLISGL (68-85)
ASAIRENEPIW (86-96)
IRENEPIWVETDRLSF (89-104)
SFLGWRHENY (103-112)
LGWRHENY (105-112)
VERYHVQTSNW (117-127)
QRAFQKLRNCKTHNDL (133-148)
QRAFQKLRNCKTHNDLINT (133-151)
INTLTRL (149-155)
IQEISGYDRVM (156-166)
YDRVMIYQFDPEWNG (162-176)
YQFDPEWNG (168-176)
ESVRQLFTSM (181-190)
ESVRQLFTSML (181-191)
SVRQLFTSM (182-190)
SVRQLFTSML (182-191)
FTSMLNHHFPASDIPAQA (187-204)
LNHHFPASDIPAQA (191-204)
LNHHFPASDIPAQARA (191-206)
NHHFPASDIPAQA (192-204)
YSINPIRIIPDVN (208-220)
YSINPIRIIPDVNAEPQPLHM (208-228)
YSINPIRIIPDVNAEPQPLHMIHKPQNTEA (208-237)
SINPIRIIPDVNAEPQPLHM (209-228)
PIRIIPDVNAEPQPLHM (212-228)
VNLSSGVLRAVSPLHMQYLRNFGVS (238-262)
RAVSPLHMQY (246-255)
QYLRNFGVS (254-262)
ASTSIGIFNE (263-272)
ASTSIGIFNEDEL (263-275)
WGIVACHHTKPRAIGRRIRRL (276-296)
WGIVACHHTKPRAIGRRIRRLLV (276-298)
WGIVACHHTKPRAIGRRIRRLLVRT (276-300)
WGIVACHHTKPRAIGRRIRRLLVRTVE (276-302)
FAAERL (303-308)
D-rel
Incubation Time (sec)
10
45
180
900
3600

Supplementary Figure 1. Individual deuterium incorporation plots of all evaluated peptides and comparison of common *Is*PadC^{PG} and *Is*PadC peptides. Peptide sequences and their corresponding positions are shown on top of each sub-panel. The relative deuterium incorporation is plotted against the labeling time in seconds. *Is*PadC dark-state traces are colored in black, *Is*PadC^{PG} monomer^{Z} dark-state traces are colored in blue, and *Is*PadC^{PG} dimer$^{E/E}$ dark-state traces are colored in red. D_{rel} values are shown as the mean of three independent measurements and error bars correspond to the sample standard deviation. The lower part of each sub-panel shows software-estimated abundance distributions of individual deuterated species on a scale from undeuterated to all exchangeable amides deuterated. Please zoom in on the electronic version to visualize all details.

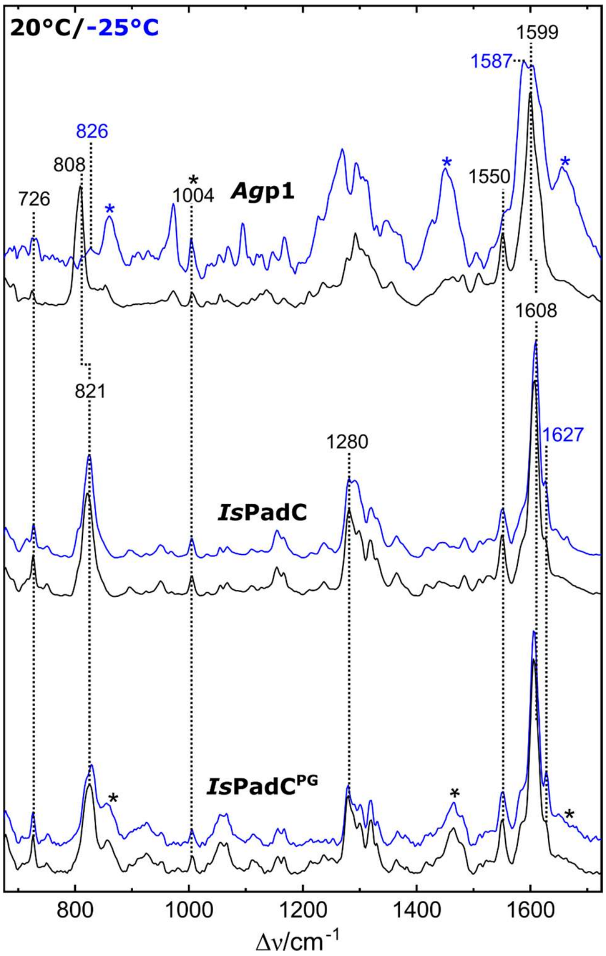

Supplementary Figure 2: RR spectra of the photoproducts of the *Is*PadC variants at different temperatures. The respective samples in the Pr state were illuminated with a 660 nm LED at T=-25°C (blue lines) and at T=20°C (black lines). All RR spectra were measured at -140°C. Unconverted Pr contributions were subtracted from the spectra. Top to bottom: *Ag*p1, *Is*PadC and *Is*PadC^{PG}. Contributions from the protein backbone and amino acid side chains are marked with asterisks (e.g. Phe-breathing mode at 1004 cm^{-1}).

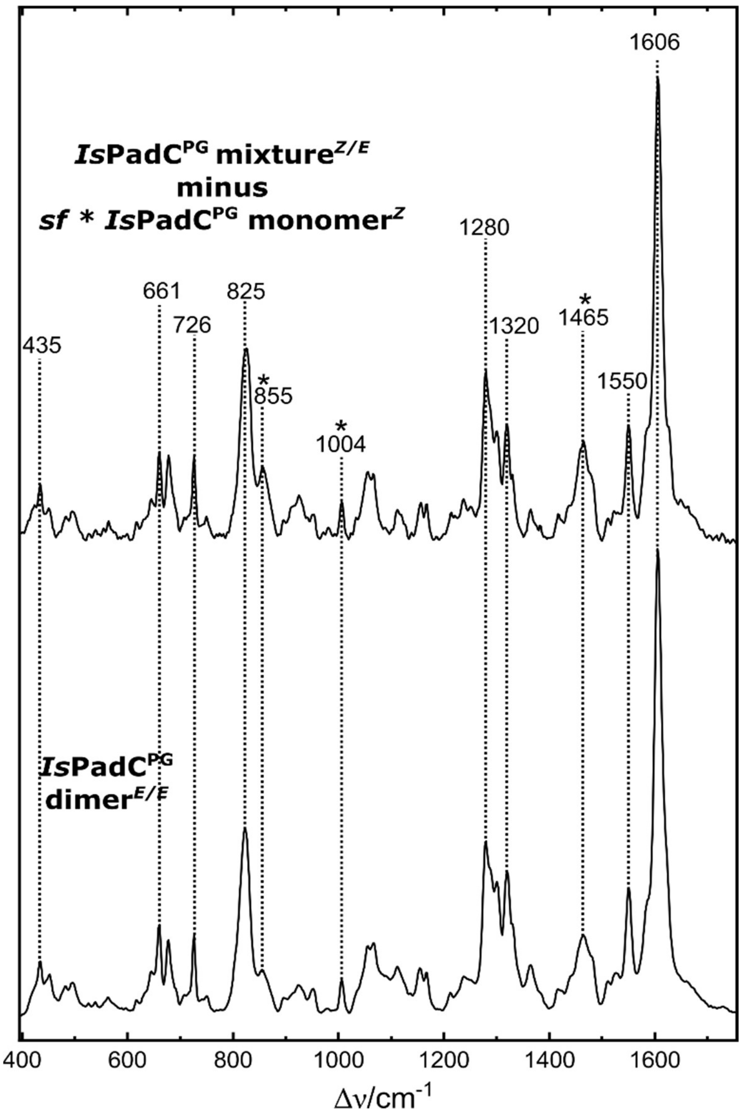

Supplementary Figure 3: RR spectra of the PG construct photoproducts obtained under different experimental conditions. The *Is*PadC^{PG} dimer$^{E/E}$ purified by gel filtration (bottom) and the photoproduct spectrum of the monomer fraction obtained at 20°C after subtraction of the dark contribution (top). Contributions from the protein backbone and buffer are marked with asterisks.

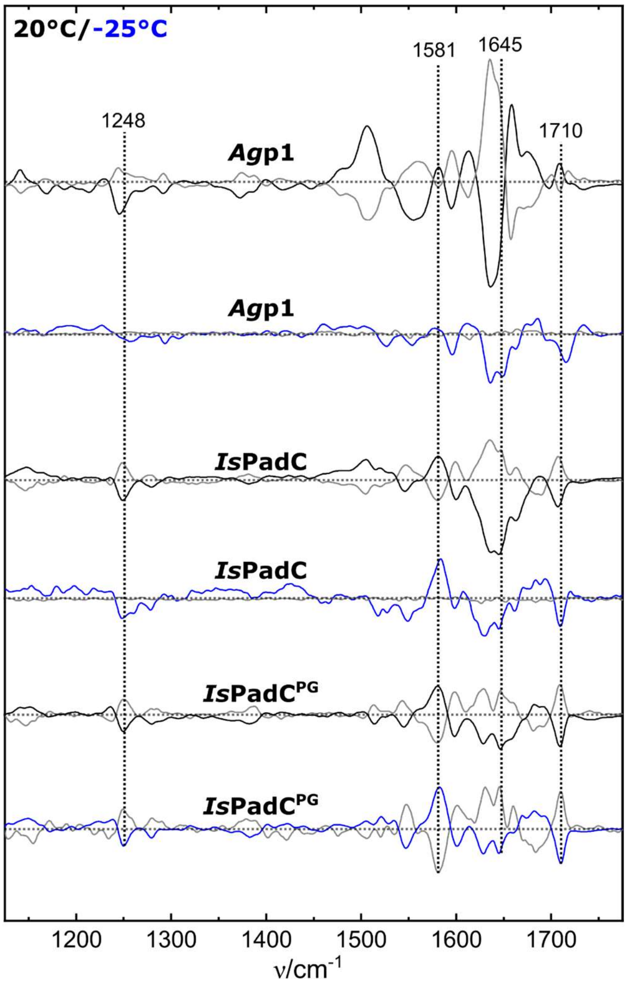

Supplementary Figure 4: Photoinduced FTIR difference spectra of *Is*PadC variants at different temperatures. Positive bands represent the respective photoproduct and negative bands the Pr state. Spectra were recorded at T = -25 °C (blue lines) and T = 20 °C (black lines). The grey traces indicate that the photoreaction was reversible (mirror image) at the respective temperature by illumination of the sample with a 750 nm diode array. Top to bottom: *Ag*p1, *Is*PadC and *Is*PadC^{PG}.

Supplementary Table 1. Crystallographic data collection and refinement statistics.

	*Is*PadC^{PG} monomer^{Z} (PDB:6SAX)*	*Is*PadC^{PG} dimer$^{E/E}$ (PDB:6SAW)*
**Data collection**		
Wavelength, Å	0.8731	1.0047
Space group	P 6_1	R 3
Cell dimensions		
a, *b*, *c* (Å)	149.42, 149.42, 77.26	343.95, 343.95, 92.40
α, β, γ (°)	90, 90, 120	90, 90, 120
Resolution (Å)	60.00 – 2.40 (2.50 - 2.40)$	60.00 – 3.00 (3.10 – 3.00)$
R_{meas} (%)	20.2 (161.8)	28.6 (139.2)
$I / \sigma I$	9.69 (1.57)	4.94 (1.12)
CC (1/2)	99.6 (55.9)	95.6 (26.8)
Completeness (%)	99.7 (99.5)	99.5 (99.4)
Redundancy	10.15 (10.42)	3.48 (3.40)
**Refinement**		
Resolution (Å)	53.89 – 2.40	58.06 – 3.00
No. reflections	38,885	81,534
R_{work} / R_{free}	0.198 / 0.236	0.197 / 0.229
No. atoms		
Protein	4,998	19,952
Ligand/ion	86 / 0	344 / 8
Water	110	8
B-factors		
Protein	49.1	67.1
Ligand/ion	38.7	67.5
Water	41.6	68.4
R.m.s. deviations		
Bond lengths (Å)	0.006	0.006
Bond angles (°)	0.818	0.865

*1 crystal has been used for data collection. $Values in parentheses are for the highest-resolution shell.

Supplementary Table 2. Overview of oligonucleotides and buffers. (a) Oligonucleotides used in this study. **(b)** Buffers used for purification and storage of the different *Is*PadC variants.

Panel **a** - oligonucleotides	
**Desired construct**	**Oligonucleotide (5'-3')**
*Is*PadC^{PG}	fw: CAGAACGTCTGTGGCTGATTCATTAAGCGGCCGCAC<br>rv: ATGAATCAGCCACAGACGTTCTGCTGCAAATTCAACGGTACG

Panel **b** – buffer systems	
**Use**	**Buffer composition**
Storage buffer	10 mM HEPES pH 7, 0.5 M NaCl, 2 mM $MgCl_2$
Storage buffer for crystallization	10 mM HEPES pH 7, 0.5 M NaCl, 2 mM $MgCl_2$
Lysis buffer	50 mM HEPES pH 7, 0.5 M NaCl, 2 mM $MgCl_2$, 10 mM imidazole
Dialysis buffer	50 mM HEPES pH 7, 0.5 M NaCl, 2 mM $MgCl_2$
